# Using Gamification of Smart Healthcare among Individuals with Bipolar Disorder

**DOI:** 10.1192/j.eurpsy.2023.462

**Published:** 2023-07-19

**Authors:** F.-H. Cheng, Y.-H. Lin, E. C.-L. Lin

**Affiliations:** Nursing, National Cheng Kung University, Tainan, Taiwan, Province of China

## Abstract

**Introduction:**

Bipolar disorder (BD) is a severe psychotic disease repeats depression, hypomania or mania. Using mobile applications to record emotions can help BD patients to self-manage and reduce emotional symptoms. Gamification applied in health-manage applications can improve the using frequency and satisfaction. Nurturing and horticultural therapy could increase the using frequency and alleviate the depression and anxiety.

**Objectives:**

This study chose plants-nurturing to add to a self-management application, and explored the users’ experiences.

**Methods:**

A one-group pretest-posttest design with qualitative interview was used. Analysis included the frequency of usage, emotional changes, and users’ feedback of the plants-nurturing in the first three months and after three months.。

**Results:**

A total of 26 participants were included. In the frequency of usage, the times and ratio of days were increased but no significant difference. The emotional symptoms were no significant difference. Positive experiences were novelty and interesting, while negative experiences were the slow rate of growth.

**Table 1.** Demographics (N=26)

	**Range**	**Mean (SD)**
**Age**	20-62	37.0(11.35)
	**n**	**Percentage**
**Gender**		
Female	15	57.7
**Educational level**		
University	14	53.8
**Employment situation**		
Employment	14	53.8

**Image:**

**Figure:**
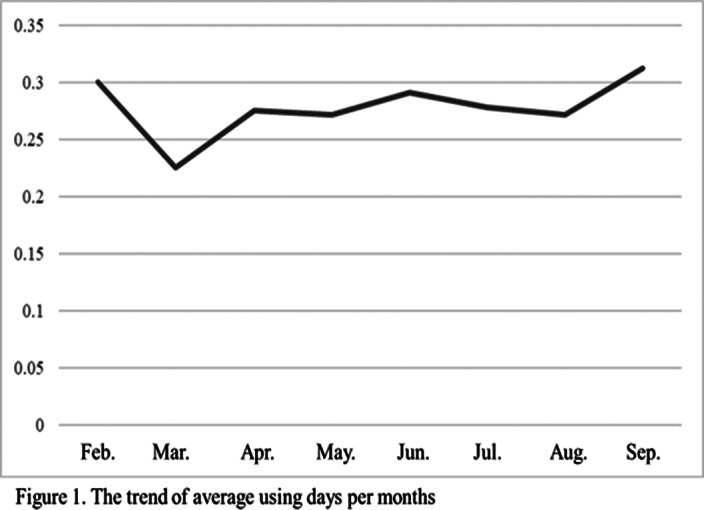

**Image 2:**

**Figure:**
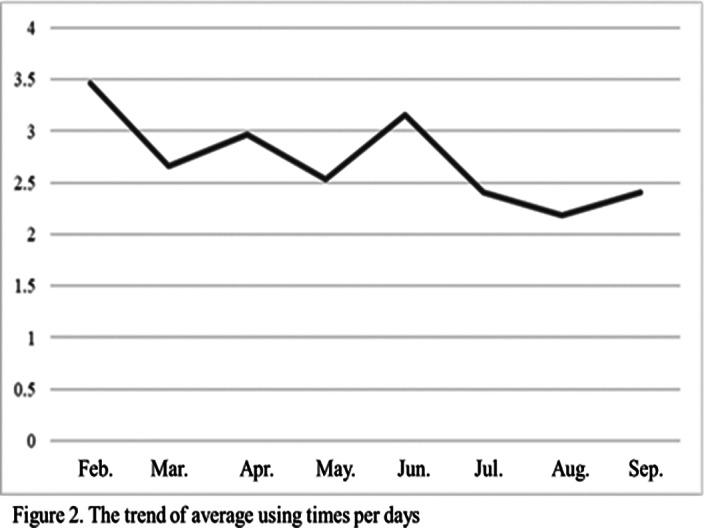

**Image 3:**

**Figure:**
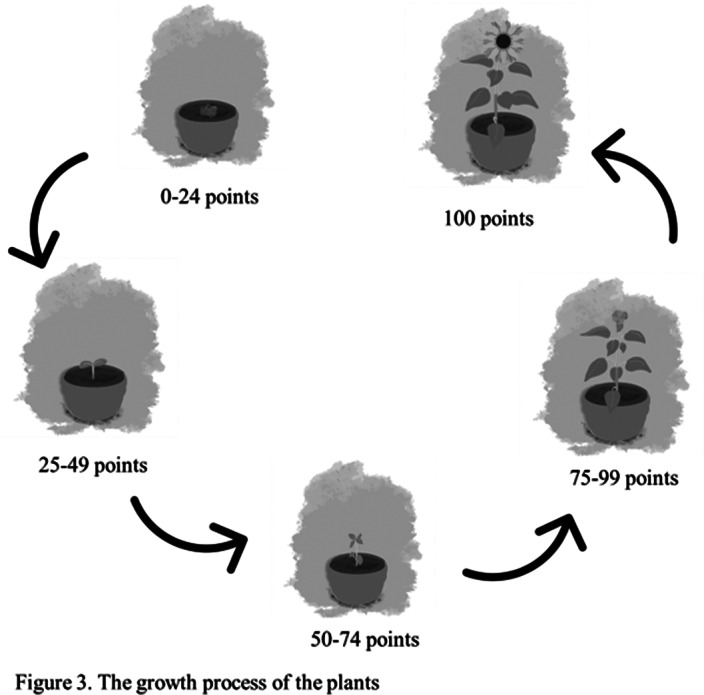

**Conclusions:**

There was a preliminary increase when adding plants-nurturing to the self-manage application, whereas the effect should be examined by further research. The more delicate elements of gamification could be included in self-manage application, considered the users’ other sensory perception in the future. Meanwhile, to improve the frequency of usage and self-management in BD patients, the subjective experiences should be explored in-depth.

**Disclosure of Interest:**

None Declared

